# Influence of the COVID-19 Pandemic on Medical Management and on Healthcare Delivery of Immune-Mediated Rheumatic and Musculoskeletal Diseases during the First Pandemic Period February to July 2020: A Systematic Review

**DOI:** 10.3390/medicina60040596

**Published:** 2024-04-04

**Authors:** Marco Schlegel, Stefan Bachmann

**Affiliations:** 1 Department of Rheumatology, Rehabilitations Zentrum Valens, Kliniken Valens, 7317 Valens, Switzerland; 2 Department of Geriatrics, Inselspital, Bern University Hospital, Faculty of Medicine, University of Bern, 3010 Bern, Switzerland

**Keywords:** COVID-19, pandemic, influence, musculoskeletal diseases, management

## Abstract

(1) *Background and Objectives*: The COVID-19 pandemic influenced the management of patients with immune-mediated rheumatic and musculoskeletal diseases (imRMDs) in various ways. The goal of our systematic review was to determine the influence of the first period of the COVID-19 pandemic (February 2020 to July 2020) on the management of imRMDs regarding the availability of drugs, adherence to therapy and therapy changes and on healthcare delivery. (2) *Materials and Methods*: We conducted a systematic literature search of PubMed, Cochrane and Embase databases (carried out 20–26 October 2021), including studies with adult patients, on the influence of the COVID-19 pandemic on the management of imRMDs. There were no restrictions regarding to study design except for systematic reviews and case reports that were excluded as well as articles on the disease outcomes in case of SARS-CoV-2 infection. Two reviewers screened the studies for inclusion, and in case of disagreement, a consensus was reached after discussion. (3) *Results*: A total of 5969 potentially relevant studies were found, and after title, abstract and full-text screening, 34 studies were included with data from 182,746 patients and 2018 rheumatologists. The non-availability of drugs (the impossibility or increased difficulty to obtain a drug), e.g., hydroxychloroquine and tocilizumab, was frequent (in 16–69% of patients). Further, medication non-adherence was reported among patients with different imRMDs and between different drugs in 4–46% of patients. Changes to preexisting medication were reported in up to 33% of patients (e.g., reducing the dose of steroids or the cessation of biological disease-modifying anti-rheumatic drugs). Physical in-office consultations and laboratory testing decreased, and therefore, newly implemented remote consultations (particularly telemedicine) increased greatly, with an increase of up to 80%. (4) *Conclusions*: The COVID-19 pandemic influenced the management of imRMDs, especially at the beginning. The influences were wide-ranging, affecting the availability of pharmacies, adherence to medication or medication changes, avoidance of doctor visits and laboratory testing. Remote and telehealth consultations were newly implemented. These new forms of healthcare delivery should be spread and implemented worldwide to routine clinical practice to be ready for future pandemics. Every healthcare service provider treating patients with imRMDs should check with his IT provider how these new forms of visits can be used and how they are offered in daily clinical practice. Therefore, this is not only a digitalization topic but also an organization theme for hospitals or outpatient clinics.

## 1. Introduction

In 2019, the novel coronavirus “Severe Acute Respiratory Syndrome-Corona Virus 2 (SARS-CoV-2)” was identified in China [1]. Coronaviruses have led to several critical disease outbreaks in the past. Important to mention are the outbreak of severe acute respiratory syndrome (SARS) in China in 2002 and of middle east respiratory syndrome (MERS) on the Arabian Peninsula in 2012 and in Korea in 2015. While these former disease outbreaks were geographically localized, SARS-CoV-2 spread rapidly over countries, causing a worldwide pandemic [2]. The disease caused by SARS-CoV-2 was subsequently named coronavirus disease 19 (COVID-19) [3,4]. At the beginning of the pandemic, the focus was primarily on an infection of the lungs with pneumonia and pneumonitis occurring, as well as the associated problems in severe cases such as acute respiratory distress syndrome (ARDS). By now, COVID-19 is known to be a multisystem infectious disease that affects different organ systems [5]. The course of COVID-19 ranges from mild to severe and critical cases (depending on risk factors) often requiring intensive care. Risk factors for a severe disease course are cardiovascular risk factors, chronic lung diseases, male sex, age over 65 years, obesity, high-dose corticosteroid use, and immunodeficiency or immunosuppressive medication [6]. Patients with immune-mediated rheumatic and musculoskeletal diseases (imRMDs) including inflammatory arthropathies (rheumatoid arthritis, spondyloarthropathies), connective tissue diseases or vasculitis are at a higher risk of infections especially due to the use of immunosuppressive medication [7].

COVID-19 and the following pandemic have therefore raised concerns amongst rheumatologists, especially regarding immunocompromised patients. Data from 2021 show that the risk for infection with SARS-CoV-2 is not increased [7] or only slightly [8] elevated in patients with imRMDs compared to the general population, but, if infected, the risks for hospitalization or for a severe disease course and death are increased by a factor of 1.58 to 2.92 [9]. Regarding medication, most conventional synthetic (csDMARD), biological (bDMARD) and targeted synthetic disease-modifying anti-rheumatic drugs (tsDMARDs) do not seem to increase the risk of infection with SARS-CoV-2 or the risk of poor outcomes of COVID-19, the exceptions being glucocorticoids > 10 mg/day, rituximab, mycophenolate mofetil (MMF) and potentially Janus kinase inhibitors (JAKis) [9].

The disease course of patients with imRMDs and SARS-CoV-2 infection has been studied widely, but systematic reviews describing the influence of the pandemic on the treatment of imRMDs are lacking. 

The aim of this systematic review is to describe the influence of the COVID-19 pandemic on the management of imRMDs during the first wave from February 2020 to July 2020 regarding availability of drugs, adherence and changes in medications, on the access to rheumatological care and medications and on the use of other healthcare delivery forms.

## 2. Materials and Methods

This review was performed according to the Preferred Reporting Items for Systematic Reviews and Meta-Analyses (PRISMA) guidelines [10]. A comprehensive search was carried out in PubMed, Cochrane and Embase databases regarding publications from 1 December 2019 to 31 October 2021. We used specific MeSH headings and additional keywords to identify studies (see search strategy in Supplementary File S1).

We selected articles in English or German including adult patients with imRMDs that evaluated the influence of the COVID-19 pandemic on the general management of imRMDs (influence on adherence or changes in medications), on the access to rheumatologically care and medications, and on the use of other healthcare delivery forms. There were no restrictions regarding study design except for systematic reviews and case reports that were excluded as well as articles on the disease outcomes in case of SARS-CoV-2 infection.

Studies found were screened independently by two reviewers (MS, SBa) for inclusion. In the first phase, the studies were screened for title and abstract, followed by full-text screening and data extraction. In case of disagreements, a consensus was reached after discussion between the two raters. Quality rating was performed according to the Oxford Centre for Evidence-Based Medicine 2011 Levels of Evidence [11]. Covidence systematic review software (version of 2021, Veritas Health Innovation, Melbourne, Australia) [12] was used as the literature management program and Zotero (version 6, Corporation for Digital Scholarship, Fairfax, VA, USA) as reference management software [13].

Because no randomized controlled trials were published and data were very heterogeneous, no meta-analysis was performed. Out of the included studies, two clusters of “influences of COVID-19 pandemic” were formed and analyzed further regarding: (i) the influence on the medical management of imRMDs; (ii) influences on healthcare delivery regarding imRMDs.

## 3. Results

### 3.1. Study Selection

**Figure 1:**
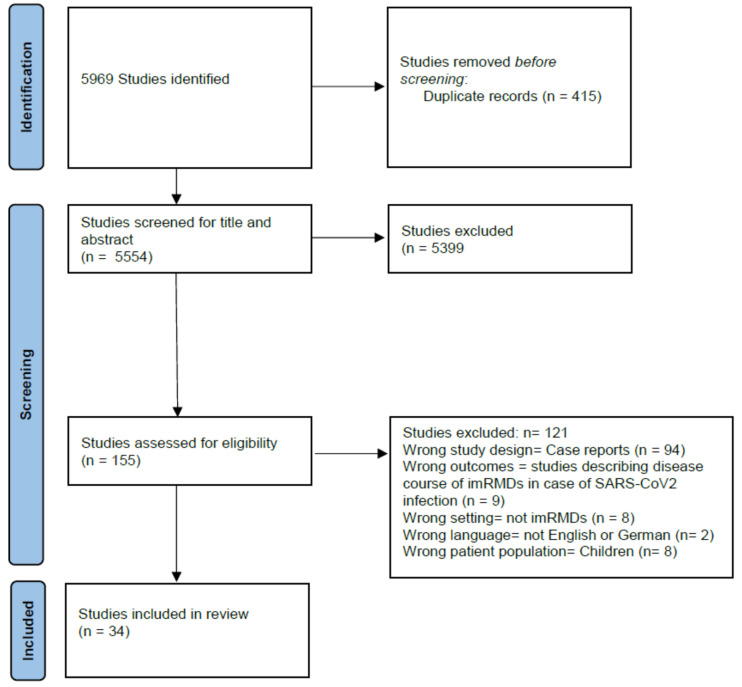
Study flow diagram.

The search strategy identified 5969 potentially relevant studies. Based on title and abstract and after removal of duplicates, 155 studies were assessed in full-text screening. A final total of 34 studies with data from 182,746 patients and from 2018 rheumatologists were included in the systematic review. Figure 1 shows the study flow according to Preferred Reporting Items for Systematic Reviews and Meta-Analyses (PRISMA) [10].

### 3.2. Study Characteristics and Levels of Evidence

The majority of included studies were surveys or questionnaires [14,15,16,17,18,19,20,21,22,23,24,25,26,27,28,29,30,31,32,33,34,35,36,37,38,39,40,41,42,43,44,45,46,47], which are evidence grade IV according to the Oxford Centre for Evidence-Based Medicine 2011 Levels of Evidence [11]. One was a cohort study, graded level III [48]. 

### 3.3. Studies Origin

Seventeen studies were from Europe [15,16,18,19,21,22,24,28,29,30,31,37,39,41,42,46,47], three from Africa [14,17,34], ten from North America [23,25,26,27,33,35,36,38,45,48,49] and five from Asia [20,32,40,43,44]. Details of the included studies are shown in Table 1. 

### 3.4. Outcomes

#### 3.4.1. Influence on the Medical Management of imRMDs

(a) Non-availability of drugs

The non-availability of rheumatic medication was a prevalent issue, important examples being hydroxychloroquine (HCQ) and tocilizumab. Shortages or difficulties in the availability of HCQ was an issue in 16–69% of patients [14,15,17,20,23,30,47], with the highest level reported from India [20]. Shortages of tocilizumab was reported in 14% of patients [45,47].

(b) Non-adherence to medication

Non-adherence to prescribed drugs was another issue. Non-adherence was very heterogeneously defined in the different studies as changing medication, the adaptation of dose or intervals without professional health advice or stopping medication or the irregular intake of medication without professional health advice. The overall non-adherence rate among all included studies was 4–46% of patients [16,26,29,30,31,32,43]. High rates of non-adherence were reported by four studies [20,21,24,33], with the highest level (46% of patients) reported from India [20]. 

A Swiss study [16] comparing the medication adherence of patients with different imRMDs before and during the pandemic found only slight adherence reductions. A significant increase in non-adherence was only seen in patients with axial spondyloarthritis (axSpA) (13% medication non-adherence in pre-COVID-19 period versus 20% during the first wave). The lowest level of medication non-adherence was reported from Denmark [21]. In this study, compliance with medication was compared between the start of the first lockdown to three months later, when society was gradually reopened. Low levels of non-adherence were reported (4–6% at the start of the lockdown versus 2–4% three months later). Further, there was a direct correlation of the incidence of SARS-CoV-2 infections in the general population and medication non-adherence: the higher the incidence of COVID-19, the lower the medication adherence [24].

(c) Drugs changed or stopped

The drugs mostly changed or stopped were bDMARDs and JAKi [28,32]. Low-dose prednisolone and csDMARDs were the least likely medications to be stopped. Longer disease durations of the underlying rheumatic disease and higher disease activity were significantly associated with medication discontinuation [19]. Disease flares were described in high proportions of the patients (63–74%) who had stopped their DMARDs [15,18]. 

Many studies reported reasons for changes in medication-taking. The following factors were significantly associated with changes in at least one medication due to patients’ fear of COVID-19 [21]: male sex (odds ratio (OR) 1.51, 95% confidence interval (95% CI) 1.21–1.89), age > 80 years compared to <39 years (OR 0.11, 95% CI 0.006–0.52), lower education (OR 0.56, 95% CI 0.45–0.69), being employed (OR 1.52, 95% CI 1.16–1.99) and the use of bDMARDs (OR 1.86, 95% CI 1.02–3.81). 

Regarding different rheumatologic diseases, a study from India [43] found that 43% of patients with inflammatory arthritis, 31% with systemic lupus erythematodes (SLE) and 13% with inflammatory myositis and scleroderma (*p* < 0.05) stopped their treatment. Further detailed information regarding non-adherence to or the non-availability of medication is shown in Table 2.

(d) Influence on the treating rheumatologist

Lastly, there was also an influence of the pandemic on the treating physician regarding medication. Rheumatologists reduced the dose of steroids in 23–36% of patients [14,47], and in 17% of patients, steroids were stopped completely [14]. In contrast, csDMARDs were stopped only rarely (in 2% of patients) [14], whereas bDMARDs were stopped more frequently (in 33% of patients) [15]. In a few cases, drug application intervals of bDMARDs were extended [28]. Moreover rheumatologists were hesitant to start a bDMARD in 75% [47] or a tsDMARD in 14% [14] of cases.

#### 3.4.2. Influences on Healthcare Delivery

(a) Avoidance of in-person visits

George et al. [27] and Banerjee et al. [36] reported the avoidance of laboratory testing in 42% and 47% of patients, respectively. Patients with imRMDs were significantly less likely to avoid in-person visits (OR 0.79 (95% CI 0.70–0.89)) or laboratory tests compared to patients with non-autoimmune RMDs (35% versus 39%, OR 0.84 (95% CI 0.73–0.96)) [26,48]. Other factors associated with the avoidance of in-person visits and laboratory testing were older age, low socioeconomic status, living in urban areas or in countries with higher COVID-19 activity and regarding medication receiving a bDMARD or JAKi [48]. 

From the patients’ perspective, high levels of unwillingness to healthcare visits were reported (21–86%) [14,15,27,30,36]. The highest levels with 86% of patients unwilling to attend the hospital were reported from Turkey [30]. An inability to communicate with or to see the rheumatologist was also frequently reported by 7% [21] to 39% [20] of patients [17,20,21,25,30].

(b) Alternative types of visits

Singh et al. [45] reported an increase in alternative types of visits to the rheumatologist related to COVID-19 compared to the pre-COVID-19 era, such as telephone visits (plus 53%), video-based Veterans Affairs Video Connect (VVC) visits (plus 44%) and clinical video tele-health (CVT) visits with a facilitator (plus 29%). Bos et al. [46] reported telephone visits to be the most commonly used form of remote consultation, with 80% of rheumatologists using exclusively telephone consultations. In-person visits were conducted only in special circumstances, such as for joint aspiration [46]. 

## 4. Discussion

This systematic review showed that COVID-19 influenced healthcare behavior in patients with imRMDs, as well as in rheumatologists and other doctors during the first pandemic wave from February to July 2020. In many cases, patients or doctors discontinued established medication. Further, the pandemic resulted in a collapse of supply chains, causing the non-availability of medication, especially in the case of HCQ and tocilizumab. Healthcare appointments took place less frequently than usual, and telehealth emerged as a solution, with remote consultations with physicians or with newly established telerehabilitation services. 

Medication non-adherence was a common problem among patients. A possible explanation could be the low availability of remote consultations at the beginning of the pandemic, resulting in feelings of insecurity with patients stopping their medication as a self-management strategy. The classes of medication that were discontinued most frequently were bDMARDs and JAKi [26,30], possibly because these immunosuppressive medications are considered the most dangerous regarding infections. 

Between different imRMDs, relevant differences in non-adherence to medication have been reported. Low numbers of non-adherence were reported in patients with vasculitis [37,38]. Patients with vasculitis are usually aware of the disease course with serious relapses in the absence of maintenance therapy, which results in an adherence to treatment [38]. Another factor increasing medical compliance is that parenteral treatments are often only possible in the hospital setting and are therefore not postponed by patients.

The sudden discontinuation of anti-rheumatic therapy is a relevant issue because it can lead to disease flares. A large proportion of patients with different imRMDs reported a flare after modifying their treatment [15,18]. This supports the recommendation of not stopping treatment during the pandemic in situations other than suspected or confirmed SARS-CoV-2 infection because resulting disease flares and higher requirements for glucocorticoids could increase the risk of SARS-CoV-2 infection [36].

The pandemic, and mostly the fear of infection with SARS-CoV-2, had a severe influence on the medication behavior of rheumatologists. A large proportion of rheumatologists reduced the dose or frequency of steroids [14,47,49], many changed DMARDs [14,22] or stopped them [14,15] and there was hesitancy to start new DMARDs [47,49]. 

N. Rebić et al. conducted a systematic review about the adherence to medication in patients with imRMDs [49]. They described non-adherence rates of 6.5–34.2% and discontinuation rates of 2–31.4% which are similar rates compared to the overall non-adherence rate of 4–46% in our systematic review. They found slightly higher numbers of physicians who reduced the dose of steroids (23–56% vs. 23–36% in our review), and they also reported of a reluctance to start bDMARDs or tsDMARDs. 

Different non-compliance rates to medical visits were reported between the different studies [22,30]. Patients with autoimmune RMDs were significantly less likely to avoid in-person visits and laboratory tests compared to patients with non-autoimmune rheumatic diseases [26,48]. These results may be explained with the fact that patients with imRMDs needed close monitoring because of their disease as well as their immunosuppressive treatment and the fear of an infection with SARS-CoV-2 was a more dominant factor determining behavior. Interestingly, a study from North America [48] reported a normalization of the rates of follow-up visits a few months after the start of the pandemic, suggesting a rapid adaptation of patients and doctors to the pandemic circumstances. 

The COVID-19 pandemic posed many challenges, but it also opened new opportunities for the development of healthcare systems. Because of the environmental risk factors for acquiring a SARS-CoV-2-infection before vaccines existed, practical steps to reduce the infection risk were introduced, including social distancing, hand hygiene and use of face masks [9]. As a consequence of social distancing, patient consultations were performed remotely whenever possible, leading to an increase in telehealth care. Prior to 2019, telehealth care was very rare or non-existent, but its use grew rapidly during the COVID-19 pandemic. Nevertheless, there were huge differences between different countries in the implementation of telehealth. Data from the USA and Australia showed an increase in telehealth, whereas data from India reported that only a small proportion of patients were aware that telehealth existed, and even fewer used it. It is likely that many patients with imRMDs, especially those from non-urban parts of emerging countries, had no access to telehealth care during the COVID-19 pandemic. A major goal of telehealth was to try to avoid disruption of healthcare and to prevent patients from stopping their medication. In addition, telephone and video-based consultations were preferred in “stable patients” with known disease courses and without the need for changing immunosuppressive medication [45]. 

Based on our personal experience, we believe that telehealth was a valuable tool to avoid disruption in healthcare and to prevent medication non-adherence in these special circumstances. However, since the pandemic period is over, the rate of telehealth consultations went back, and the advantages of telehealth and remote consultations are only seen in patients who have mobility difficulties to reach the treating physician’s office. In these situations, telehealth and remote consultations are still a valuable instrument to increase or hold adherence to medications.

### Strengths and Limitations

This study has several strengths. Although most studies were of low evidence grade (III or IV), data from 182,746 patients and 2018 interviewed rheumatologists were included. Together with the fact that studies from four different continents and different countries were included, these large numbers paint a global picture of the influence of the pandemic on imRMDs. The influence covers a broad spectrum of issues occurring during the COVID-19 pandemic, including compliance with medication, the shortage of certain medications and problems with the delivery of healthcare. In addition, new aspects are described, such as telemedicine and telerehabilitation services, which were set up as substitutes for former in-person routine care. The study also has limitations. First, regarding the heterogeneity of the reported outcomes and the study designs, it was not possible to carry out a meta-analysis and to evaluate the results statistically using odds ratios. Inhomogeneous reporting and differences in research methodology between the studies made comparisons difficult, and a generalization of the results may not be suitable. Furthermore, definitions of non-adherence with medication varied between studies. Therefore, it was demanding to extract and compare the different results rationally. Most of the included studies were surveys, which leads to some typical limitations regarding the study design. Surveys may lead to inclusion biases, as patients who are more interested or worried about COVID-19 are generally more willing to participate. The responses are self-reported and cannot be verified. Survivorship bias is also probable as very sick or deceased patients cannot participate. Further, patients with a relatively higher socioeconomic status have a greater online presence and affinity to online surveys and are therefore probably overrepresented. As many of the results were published only as case reports and congress abstracts, those results were not included in the present study. Important information may therefore have been missed. Finally, this review shows only results regarding the influence on the treatment of imRMDs of the first wave of the COVID-19 pandemic lasting February 2020 to July 2020. 

## 5. Conclusions

The COVID-19 pandemic influenced the management of patients with imRMDs, especially during the first wave from February 2020 to July 2020. The influence of the pandemic was diverse regarding adherence to medication, shortage of some medications, adherence to doctor visits or laboratory testing and governmental interventions. To preserve adherence to healthcare, the COVID-19 pandemic was a starting point for new healthcare systems. Remote and telehealth consultations were implemented. These new forms of healthcare delivery should be spread and implemented worldwide to routine clinical practice to be ready for future pandemics. Every healthcare service provider treating patients with imRMDs should check with his IT provider how these new forms of visits can be used and how they are offered in daily clinical practice. Therefore, this is not only a digitalization topic but also an organization theme for hospitals or outpatient clinics. 

## Figures and Tables

**Table 1:** Characteristics of included studies [14,15,16,17,18,19,20,21,22,23,24,25,26,27,28,29,30,31,32,33,34,35,36,37,38,39,40,41,42,43,44,45,46,47,48].

Study, Year, Country/World Region	Study Design	Level of Evidence	Research Question	Population	Specific Influence on the Treatment/Main Outcome Measures
Akintayo et al. 2021, Africa [14]	Survey	IV	To identify changes in rheumatology service during the COVID-19 pandemic in Africa	554 questionnaires completed by rheumatologists	66% described shortage of hydroxychloroquine; 36% reduced corticoids; 16% stopped corticoids; 14% avoided start of biologics.
Batibay et al. 2021, Turkey [15]	Survey	IV	“To determine how the COVID-19 pandemic affected routine care in rheumatology and if there were any changes in rheumatologic medication use”	320 patients with different immune-mediated rheumatic diseases	16% had problems in obtaining hydroxychloroquine; 12% changed medication without advice; 33% interrupted bDMARDs with advice from their physician; 9% stopped bDMARDs on their own.
Ciurea et al. 2021, Switzerland [16]	Survey	IV	Adherence to anti-rheumatic drugs before and during the first COVID-19 wave	666 patients with immune-mediated rheumatic diseases (RA, AxSpa and PsoA) compared pre-COVID and during COVID-19 wave	20% of AxSpA patients were not adherent to anti-rheumatic drugs during the first COVID wave (versus 13% pre-COVID); regarding the other diseases, only a slight non-significant increase in non-adherence was observed.
Ziadé et al. 2020, Arab countries (Levant, Gulf, North Africa) [17]	Survey	IV	Influence of the COVID-19 pandemic on access to rheumatology care for patients with chronic rheumatologic diseases	2190 patients with chronic rheumatic diseases from different Arab countries	70% reported no negative effect on medication adherence; 18% described shortage of hydroxychloroquine; thereof 15% had to stop medication due to shortage; 8% stopped medication because of fear of infections.
Costantino et al. 2021, France [18]	Survey	IV	Consequences of the pandemic on rheumatic disease management	655 patients with immune-mediated rheumatic diseases (AxSpA, RA, PsoA)	More than one-third of patients (34.2%) suspended or decreased the dosage of one drug. NSAIDs were the most commonly decreased medication (33.7% of patients), followed by bDMARDs (13.4%) and cDMARDs (10.1%).
Coskun et al. 2021, Turkey [19]	Survey	IV	To determine if patients maintained their treatment for imRMDS during the pandemic period To determine the factors responsible for discontinuation	278 patients with immune-mediated rheumatic diseases (ankylosing spondylitis (AS) and RA) from Turkey	Overall, 22% of patients reduced or stopped treatment. 27% of the patients stopped bDMARDs. 5% stopped low-dose glucocorticoids. 4% stopped methotrexate.
Ganapati et al. 2021, India [20]	Survey	IV	To describe the influence of COVID-19 on patients with chronic rheumatic diseases	1533 completed responses of patients with chronic rheumatic diseases (inflammatory and non-inflammatory) across India	47% of patients were fully compliant to medication. 35% were partly compliant and 11% discontinued their medication. 90% of patients experienced difficulty in procuring medication. 69% of patients on hydroxychloroquine had difficulty obtaining it.
Glintborg et al. 2021, Denmark [21]	Survey	IV	To explore self-protection and health behavior including adherence to disease-modifying anti-rheumatic treatment (DMARD) during the COVID-19 pandemic	12,789 patients with immune-mediated rheumatic diseases (RA, PsoA, AxSpA, connective tissue disorders)	With the beginning of the pandemic restrictions, 4% of patients changed the dosage of csDMARDs, whereas 3 months later, only 2% of patients did. With the beginning of the pandemic restrictions, 6% of patients changed the dosage of bDMARDs, whereas 3 months later, only 4% of patients did.
Glintborg et al. 2021, Denmark [22]	Survey	IV	To investigate if the pandemic affected the treat-to-target strategy as evaluated by disease activity and to evaluate access to physical consultations during the COVID-19 pandemic	7836 patients with immune-mediated rheumatic diseases (RA, PsoA or AxSpA) from Denmark	Glucocorticoid injections decreased relatively by 16% in patients with rheumatoid arthritis and by 10% in patients with psoriasis arthritis. DMARDs had been altered in dose in 13% of patients compared to before the pandemic.
Mancuso et al. 2021, USA [23]	Survey	IV	To obtain detailed information about patients’ experiences with their medications during the COVID-19 Pandemic	112 patients with immune-mediated rheumatic diseases in New York City	11–14% of respondents reported self-imposed or physician-directed changes to medications. 61% of the patients treated with HCQ had difficulties obtaining it.
Hasseli et al. 2021, Germany [24]	Survey	IV	The goal was to determine the influence of the SARS-CoV-2 lockdown on patients with immune-mediated rheumatic diseases on their adherence to immunomodulatory medication	4252 patients with immune-mediated rheumatic disease from Germany	Before the national lockdown, 4% of the patients discontinued their medication. During and after the national lockdown, reported discontinuations decreased to 2%.
Hausmann et al. 2021, USA (survey was worldwide) [25]	Survey	IV	To determine the influence of the COVID-19 pandemic on healthcare behavior of patients with immune-mediated rheumatic diseases	9300 patients with inflammatory rheumatic diseases originating from 90 countries, mostly USA and Europe	82% continued their anti-rheumatic medications as prescribed. The remaining 18% of participants treated with anti-rheumatic medications discontinued at least one of their medications.
George et al. 2021, USA [26]	Survey	IV	To better understand the concerns and behaviors of patients with autoimmune rheumatic disease compared with patients without autoimmune rheumatic diseases	2319 patients with a non-autoimmune rheumatic disease and 6885 patients with an autoimmune rheumatic disease from America	10% of patients with autoimmune rheumatic disease stopped immunomodulatory medication. Patients on biologics or JAK inhibitors were more likely to stop their medication than the other patients in the population (OR 1.53 (1.22–1.90)). Patients with autoimmune disease were significantly less likely to avoid in-person visits (predicted probability 45.2% versus 51.0%, OR 0.79) and avoid laboratory tests compared to patients with non-autoimmune rheumatic diseases (34.9% versus 38.8%, OR 0.84). Patients who had a telemedicine visit were at greater risk of stopping a medication than those with an office visit (OR 1.54).
George et al. 2021, North America [27]	Survey	IV	To learn about patient’s concerns, healthcare disruption and use of telehealth as well as interruption in DMARDs.	1517 participants with common immune-mediated rheumatic diseases (RA, PsoA, SpA or SLE) representing all 50 states of the USA	15% of the patients without COVID-19 stopped their DMARDs, mostly on their own.
Kalyoncu et al. 2021, Turkey [28]	Survey	IV	The goal was to determine the treatment adherence of patients with immune-mediated arthritis receiving b/s DMARDs	1394 patients with immune-mediated rheumatic diseases receiving either biologic or synthetic DMARDs in Turkey	18% of all patients discontinued their bDMARDs, 32% of them on recommendation of the physician, 45% on their own demand. 14% of the RA patients and 21% of the SpA patients discontinued their bDMARDs. Among patients with RA, etanercept was the least frequently discontinued bDMARD (5.4% of the patients), whereas tocilizumab was the most frequently discontinued (20.5% of the patients). Those who discontinued their bDMARDs in SpA were younger than those who did not (median age, 40 years versus median age, 44 years). 57% of the communications between doctor and patient were via phone. 77% of the patients, who were communicating with their physician, were recommended to continue bDMARD therapy.
Murray et al. 2021, Ireland [29]	Survey	IV	The goal of the survey was to assess COVID-19 status, rheumatic musculoskeletal disease diagnoses and adherence to rheumatologic medication	1381 patients with immune-mediated musculoskeletal diseases from Ireland	Adherence to anti-rheumatic medications was 84%, and 57% were using health authorities guidelines for information on medication use. In the patients who followed guidelines, adherence rates were higher (89.3% versus 79.9%). Lower adherence rates were found in those with COVID-19 symptoms (64.0% versus 85.1%).
Seyahi et al. 2020, Turkey [30]	Survey	IV	The goal was to learn about the healthcare behavior of rheumatic disease patients during COVID-19 pandemic	771 patients with rheumatic disease, and as a control group, 535 hospital workers and 917 teachers/academic staff.	Overall, 22% of the patients discontinued their medications. In patients with SpA, 54% discontinued their medication. bDMARDs were the most frequent drugs to be stopped: anti-IL-1 agents in 40%, anti-TNF agents in 35%, interferon in 33%, tocilizumab in 29%, rituximab in 7% of the patients. Prednisolone (low dose), azathioprine, methotrexate, leflunomide, colchicine and sulfasalazine were least likely to be stopped (≤10% for each drug). 11% of the patients on hydroxychloroquine had to skip or stop the drug due to shortage or failure of prescription renewal. 86% of patients with rheumatic diseases were unwilling to go to the hospital. Only 14% of patients visited the outpatient clinic “as it was before”, 43% “did not want to come” and 28% “were advised to postpone their visits”.
Sloan et al. 2021, UK [31]	Survey	IV	To assess the influence of the COVID-19 pandemic on medical care and healthcare behavior of patients with SLE and other autoimmune rheumatic diseases	111 patients with autoimmune rheumatic diseases	10% of patients reduced or stopped their medication, and 10% increased the dose of their medication without advice of their doctor. 70% reported a canceling of their appointments, tests and treatments more or much more frequently since the pandemic. Between 35% and 45% felt that care from GPs, rheumatologists and other specialists had been worse/much worse during the pandemic.
Hassen et al. 2020, Saudi Arabia [32]	Survey	IV	To understand the influence of the COVID-19 pandemic on healthcare behavior of patients with immune-mediated rheumatic diseases	637 patients with immune-mediated rheumatic disease from Saudi Arabia	Worsening disease activity perception was significantly associated with poor medication adherence. 86% were adherent and 14% were not adherent to their anti-rheumatic medication. 30% altered their prescribed medication(s) either by decreasing, increasing or interrupting the dosage. 48% had trouble obtaining their medication during COVID-19 outbreak.
Guaracha -Basanez et al. 2021, Mexico [33]	Survey	IV	To determine the influence of the pandemic on healthcare interruption on the clinical status of the underlying rheumatic disease	670 patients with rheumatic diseases (mainly SLE and RA) in Mexico City	60% of patients were found to be compliant with the prescribed treatment. 51% experienced healthcare interruptions.
Abualfadl et al. 2020, Egypt [34]	Survey	IV	To determine the influence of the COVID-19 pandemic on patients with RA	1037 patients with RA aged 18 years and older	The following percentage of patients had difficulties obtaining their anti-rheumatic medication: hydroxychloroquine/chloroquine (42%), methotrexate (6%), biologics (2%) and leflunomide (1%).
Michaud et al. 2020, USA [35]	Survey	IV	To assess the influence of COVID-19 pandemic on medical treatment of patients with RA in the USA	734 patients with RA in the USA	30% of patients reported medication changes. Changers more commonly used glucocorticoids (33% versus 18%) and less commonly non-hydroxychloroquine conventional DMARDs (49% versus 62%) in the pre-COVID era. While JAK inhibitor use was associated with change (OR 1.9), only pre-COVID glucocorticoids remained a strong predictor for changes (OR 3.0). bDMARDs and JAK inhibitor users reported stopping or delaying the intake of that DMARD more often than users of csDMARDs or hydroxychloroquine (16–18% versus 4–8%). Overall, 4–7% could not obtain their medication. 10% of hydroxychloroquine users could not obtain it. Percentage of respondents who cancelled or postponed appointments was between 28% and 35%. 42% and 47%, respectively, of patients on non-TNF bDMARD or JAK inhibitors reported switching to telehealth appointments. 34–36% of patients on hydroxychloroquine and other csDMARDs reported switching to telehealth. 31% of patients in the TNF bDMARD group reported switching to telehealth.
Banerjee et al. 2020, USA [36]	Survey	IV	To identify effects of COVID-19 pandemic on patients with vasculitis and especially their health related behavior	662 patients with vasculitis	11% of all patients stopped their immunosuppressive therapy. 8% of patients temporarily discontinued rituximab. 13% reported avoiding receiving an infusion with rituximab. 6% of patients on <10 mg prednisone equivalent/day and 11% > 10 mg prednisone equivalent/day stopped their medication. 66% of all patients avoided doctor’s visits. 47% of all patients avoided laboratory tests. 46% of all patients had a telehealth visit.
Ince et al. 2021, Turkey [37]	Survey	IV	To determine the influence of COVID-19 pandemic on disease activity and medical treatment of patients with vasculitis	103 patients with vasculitis living in Turkey	32% of patients missed at least one outpatient appointment. Attendance rate for appointments was higher among patients who used parenteral treatment in comparison to oral treatment. 5% of patients were non-compliant to their medication.
Kant et al. 2021, USA [38]	Survey	IV	To determine the influence of the COVID-19 pandemic on the treatment and disease course of patients with ANCA-associated vasculitis	206 patients with ANCA-associated vasculitis from two centers (Baltimore (USA) and Lancashire (UK))	7% reduced medication dosage. 10% postponed maintenance rituximab infusion. 0% had regular in-person visit with physician. 69% had video visits with physician. 13% rescheduled clinic visits. 16% decreased blood collection frequency.
Cornet et al. 2021, Europe [39]	Survey	IV	Availability of hydroxychloroquine during the first wave of COVID-19 pandemic	2075 patients with lupus during the first wave of the COVID-19 pandemic registered in the lupus Europe’s patient advisory network	48% could obtain hydroxychloroquine from the first place they asked, 11% could obtain the drug by going to more than one pharmacy. 9% could not obtain any hydroxychloroquine during the first wave of the pandemic. During the second wave, only 0.8% of patients could not obtain any hydroxychloroquine.
Rathi et al. 2021, India [40]	Survey	IV	To assess the influence of COVID-19 pandemic on the treatment of SLE	1040 patients with SLE	36% of patients reported problems in availability of drugs due to lockdown. Of these, 40% of patients needed to change their medication due to non-availability. 1% of patients had missed their scheduled cyclophosphamide dose due to non-availability of drug or problems to attend outpatient clinics for intravenous infusion. 22% of patients faced difficulty in the availability of hydroxychloroquine. 54% of patients missed their scheduled follow-up visits. 37% of patients were unable to obtain their diagnostics carried out due to closure of laboratories and hospitals.
Gupta et al. 2021, England [41]	Survey	IV	To determine the influence of the COVID-19 pandemic on the healthcare of systemic scleroderma patients	291 patients with scleroderma from all over the world	15.1% of the patients on steroids required an increase in dose in the current situation. 38.1% of respondents faced hurdles in procuring medicines. Of the 14.4% that were on infusions. 45% had to delay it. Physiotherapy sessions were disrupted in 25%. 7% could not contact their specialist. Another 24% experienced difficulty contacting their specialist.
Gupta et al. 2020, England [42]	Survey	IV	To determine the influence of the COVID-19 pandemic on the healthcare of patients with myositis	608 patients with myositis from all over the world (mostly USA and England)	26% of patients faced hurdles in procuring medicines. 25% of the included patients were due for infusions, 22% of which had to delay treatment and 7% were still searching for an alternative. Of the patients who faced difficulty in obtaining their medication, 10% were forced to stop treatment. 26% experienced difficulty in contacting their specialist, and 5% were unable to do so.
Kavadichanda et al. 2020, India [43]	Survey	IV	To evaluate the feasibility of having teleconsultation among the socioeconomically marginalized sections of the society in India and to determine the influence on medical treatment	373 patients from India with rheumatologic musculoskeletal diseases	69% of patients continued the drugs based on previous prescriptions, and 31% stopped them abruptly. 43% of patients with immune-mediated arthritis stopped their treatment abruptly compared to 31% of patients with SLE (31%) and 13% of patients with inflammatory myositis or scleroderma. 90% found tele-rheumatology consultation easy. 76% considered that tele-rheumatology was better than in-person consultation in circumstances of the pandemic. 16% felt that tele-rheumatology was not as good as in-person visits.
Kavadichanda et al. 2021, India [44]	Survey	IV	To evaluate the influence of COVID-19 pandemic on access to healthcare of systemic sclerosis patients in India	336 patients with systemic sclerosis from India	Scheduled outpatient visit was missed by 92% of the patients. 22% skipped drugs. 53% missed laboratory testing. 24% faced problems with availability of medicines.
Singh et al. 2020, USA [45]	Survey	IV	To assess the experience, views and opinions of rheumatology providers during the COVID-19 pandemic	103 rheumatologists answering a survey	32% of responders reported a medication shortage. Shortage of hydroxychloroquine was reported in 45%, of IL-6 inhibitors in 15%, non-TNF biologics in 1%, Janus inhibitors in 1%. An increase in 50% or more in the following types of visits related to COVID-19 were reported: “(1) telephone visits, 53%; (2) video-based VA video connect (VVC) visits, 44%; and (3) clinical video tele-health (CVT) visits with a facilitator, 29%”.
Bos et al.2020, the Netherlands [46]	Survey	IV	To determine the influence of COVID-19 pandemic on the delivery of care using telemedicine for patients with rheumatic musculoskeletal disease from the perspective of rheumatologists in the Netherlands	75 members of the Dutch rheumatologist society were interviewed during 8–22 of May 2020	99% of the rheumatologists used telephones and 9% used video consultations. More than 80% of the outpatient consultations were performed exclusively via telephone.
Dejaco et al. 2020, Austria [47]	Survey	IV	To assess how the COVID-19 pandemic has affected decisions of rheumatologists	1286 rheumatologists were questioned in 58 countries	82% of rheumatologists indicated cancellation or postponement of face-to-face visits of new patients. 91% indicated cancellations/postponements in follow-up patients (with 96% offering remote consultation). 74% of rheumatologists indicated bDMARDs/tsDMARDs were less likely to be started during pandemic. 49% reported shortage of HCQ and consequently it had to be stopped in 10% of patients. 14% reported shortage of tocilizumab. 15% recommended decreasing and 2% stopping NSAID in asymptomatic patients. 23% recommended to decrease and 0.1% to stop glucocorticoids in asymptomatic patients
George et al. 2021, USA [48]	Cohort study	III	Examination of trends in in-person versus telehealth visits versus cancelled visits during COVID-19 pandemic	126,550 patients extracted from the analytic cohort from the Columbus electronic health record data warehouse of the American Arthritis and Rheumatology Associates network during the year 2020	Overall follow-up visit volume decreased by 25% in the COVID-19 period but rebounded within a few months to pre-COVID-19 levels. Telehealth visits pre-COVID-19 were nearly non-existent and increased to 41% and 28% of all follow-up clinician visits in the COVID-19 period and after the first wave of the pandemic. 90% of telehealth visits were video-based, 7% via phone and 2% digital. Up to maximum of 60% of visits were cancelled during the COVID-19 transition period. In the COVID-19 period in 2020, the odds of starting a new biologic or JAKi therapy for an RA patient was substantially lower (adjusted odds ratio = 0.55) compared to the corresponding 6-week period in 2019.

ARD = autoimmune rheumatic disease; AxSpA = axial spondyloarthritis; bDMARDs = biological disease-modifying anti-rheumatic drugs; CQ = chloroquine; csDMARDs = conventional synthetic disease-modifying anti-rheumatic drugs; GPs = general practitioners; HCQ = hydroxychloroquine; NSAID = non-steroidal anti-inflammatory drug; OR = odds ratio; PsoA = psoriasis arthritis; RA = rheumatoid arthritis; SLE = systemic lupus erythematosus; SpA = spondyloarthritis; tsDMARDs = targeted synthetic disease-modifying anti-rheumatic drugs.

**Table 2:** Rates of non-adherence and of difficulties obtaining medication regarding different immune-mediated RMDs.

Disease	Non-Adherence to or Discontinuation of Medication (% of Patients) *	Difficulties Obtaining Medication (% of Patients) *
**Inflammatory arthritis**		
Rheumatoid arthritis	14% (bDMARDs) [27] 19% [29] 22% (DMARDs) [15] 23% [17] 25% [18]	42% (hydroxychloroquine) [33] 10% (hydroxychloroquine) [34] 4–7% [34]
**Vasculitis**		
Vasculitis in general	5% [36] 11% [35] 20% [29]	No information
**Spondyloarthritis**		
Spondyloarthritis in general	19% [18] 20% (DMARDs) [15] 21% (bDMARDs) [27] 38% [17] 54% [29]	No information
Psoriasis arthritis	19% (DMARDs) [15] 31% [17]	No information
**Myositis**		
Myositis in general	No information	26% [41] (myositis)
**Connective tissue diseases**		
Overall	14% [29]	
SLE	No information	52% (hydroxychloroquine) [38] 29% (hydroxychloroquine) [39] 36% [39]
Systemic sclerosis	22% [43]	38% [40] 24% [43]
**Periodic fever syndromes**		
Familial Mediterranean fever	15% [29]	No information

* If the number relates to a specific drug, it is mentioned in brackets. Otherwise, the number relates to non-adherence, discontinuation or difficulties obtaining medication in general (numbers in square brackets = literature reference numbers).

## Data Availability

For original data reported in this systematic review, we refer to the original publications.

## Supplementary Materials

The following are available online at https://www.mdpi.com/article/10.3390/medicina60040596/s1, Supplementary File S1: Search Strategy.

## Abbreviations

axSpA	axial spondyloarthritis
csDMARDs, bDMARDs, tsDMARDs	conventional synthetic, biological and targeted synthetic disease-modifying anti-rheumatic drugs
CVT	clinical video telehealth
HCQ	hydroxychloroquine
imRMDs	immune-mediated Rheumatic and musculoskeletal diseases
JAKi	Janus kinase inhibitors
MMF	mycophenolate mofetil
NSAIDs	non-steroidal anti-inflammatory drugs
PRISMA	Preferred Reporting Items for Systematic Reviews and Meta-Analyses
PsoA	psoriasis arthritis
RA	rheumatoid arthritis
SLE	systemic lupus erythematodes
VVC	video-based Veterans Affairs Video Connect
