# Supplementary material for: Influence of the COVID-19 Pandemic on Medical Management and on Healthcare Delivery of Immune-Mediated Rheumatic and Musculoskeletal Diseases during the First Pandemic Period February to July 2020: A Systematic Review
Source: Medicina (Kaunas). 2024 Apr 4;60(4):596. doi: 10.3390/medicina60040596 (PMC11052197; doi:10.3390/medicina60040596)
Supplement: Supplementary file 1 [file medicina-60-00596-s001.zip › medicina-2870314-supplementary.pdf]

Supplementary File **S1- Search Strategy**

Search-terms according to PICO :

Search-Terms on Embase :

	**All fields**	**Emtree-Terms**
**Patient**	Musculoskeletal Diseases OR Musculoskeletal Disorders OR Bone Diseases OR Fasziitis Plantaris OR Joint Diseases OR Arthritis OR Halux Rigidus OR Joint Deformities OR Synovitis OR Muscular Diseases OR Contracture OR Fibromyalgia OR Chronic Fatigue Syndrom OR Muscular Disorders OR Myofascial Pain Syndromes OR Myopathies OR Myositis OR Inclusion Body Myositis OR Polymyositis OR Dermatomyositis OR Polymyalgia Rheumatica OR Tendinopathy OR Rheumatoid Arthritis OR Crystal Arthropathies OR Gout OR Chondrocalcinosis OR Osteoarthritis OR Rheumatic Fever OR Sjogren’s syndrome OR Adult-onset Still’s Disease OR Felty Syndrom OR Vasculitis OR Aortitis OR Arteritis OR Giant Cell Arteritis OR Polyarteritis Nodosa OR Takayasu Arteritis OR Behcet Syndrom OR Purpura Schoenlein-Henoch OR Anti-Neutrophil Cytoplasmic Antibody-Associated Vasculitis OR Churg-Strauss Syndrom OR Granulomatosis With Polyangiitis OR Microscopic Polyangiitis OR Rheumatoid Vasculitis OR Thromboangiitis Obliterans OR Cutaneous Leukocytoclastic Vasculitis OR Spondylarthritis OR Psoriatic Arthritis OR Reactive Arthritis OR Ankylosing Spondylitis OR Inflammatory Bowel Disease-Associated Arthritis OR Infectious Arthritis OR Whipple Disease OR Hereditary	'musculoskeletal disease'/exp OR 'musculoskeletal disorders'/exp OR 'bone disease'/exp OR 'fasciitis'/exp OR 'musculoskeletal system inflammation'/exp OR 'bursitis'/exp OR 'chondritis'/exp OR 'enthesitis'/exp OR 'synovitis'/exp OR 'arthropathy'/exp OR 'arthritis'/exp OR 'muscle disease'/exp OR 'myofascial pain'/exp OR 'myopathy'/exp OR 'myositis'/exp OR 'antisynthetase syndrome'/exp OR 'dermatomyositis'/exp OR 'inclusion body myositis'/exp OR 'polymyositis'/exp OR 'rheumatic polymyalgia'/exp OR 'tendinitis'/exp OR 'rheumatoid arthritis'/exp OR 'gout'/exp OR 'crystal arthropathy'/exp OR 'chondrocalcinosis'/exp OR 'osteoarthritis'/exp OR 'rheumatic fever'/exp OR 'Sjoegren syndrome'/exp OR 'disorders of connective tissue metabolism'/exp OR 'ankylosing spondylitis'/exp OR 'ankylosing spondylitis'/exp OR 'Behcet disease'/exp OR 'lupus erythematosus'/exp OR 'Reiter syndrome'/exp OR 'scleroderma'/exp OR 'localized scleroderma'/exp OR 'systemic sclerosis'/exp OR 'Sjoegren syndrome'/exp OR 'adult onset Still disease'/exp OR 'Felty syndrome'/exp OR 'vasculitis'/exp OR 'ANCA associated vasculitis'/exp OR 'arteritis'/exp OR 'brain vasculitis'/exp OR 'Buerger disease'/exp OR

	Autoinflammatory Diseases OR Cryopyrin-Asssociated Periodic Syndrome OR Familial Mediterranean Fever OR systemic scleroderma OR diffuse scleroderma OR Sarcoidosis OR Antiphospholipid Syndrom OR Undifferentiated Connective Tissue Diseases OR Antisynthetase Syndrom OR Anti-Glomerular Basement Membrane Disease OR Systemic Lupus Erythematosus OR Osteoporosis OR Bone Density	'capillaritis'/exp OR 'Churg Strauss syndrome'/exp OR 'granulomatous vasculitis'/exp OR 'large vessel vasculitis'/exp OR 'leukocytoclastic vasculitis'/exp OR 'microscopic polyangiitis'/exp OR 'pulmonary vasculitis'/exp OR 'rheumatoid vasculitis'/exp OR 'small vessel vasculitis'/exp OR 'systemic vasculitis'/exp OR 'vasculitic rash'/exp OR 'endarteritis'/exp OR 'giant cell arteritis'/exp OR 'necrotizing arteritis'/exp OR 'periarteritis'/exp OR 'polyarteritis'/exp OR 'polyarteritis nodosa'/exp OR 'temporal arteritis'/exp OR 'temporal arteritis'/exp OR 'Wegener granulomatosis'/exp OR 'psoriatic arthritis'/exp OR 'aortic arch syndrome'/exp OR 'anaphylactoid purpura'/exp OR 'leukocytoclastic vasculitis'/exp OR 'spondylarthritis'/exp OR 'reactive arthritis'/exp OR 'infectious arthritis'/exp OR 'intestine lipodystrophy'/exp OR 'autoinflammatory disease'/exp OR 'cryopyrin-associated periodic syndrome'/exp OR 'familial Mediterranean fever'/exp OR 'sarcoidosis'/exp OR 'antiphospholipid syndrome'/exp OR 'undifferentiated connective tissue disease'/exp OR 'glomerulonephritis'/exp OR 'lupus erythematosus'/exp OR 'metabolic bone disease'/exp OR 'bone demineralization'/exp OR 'osteoporosis'/exp OR 'osteomalacia'/exp OR 'osteopenia'/exp
**Intervention**	COVID-19 OR Severe Acute Respiratory Syndrome Coronavirus 2 OR SARS-CoV2 OR COVID-19 pandemic OR	Coronavirus disease 2019 OR severe acute respiratory syndrom coronavirus 2

	Novel coronavirus pneumonia OR SARS-CoV2-Infection OR COVID-19 Virus Disease OR COVID-19 Virus Infection OR Coronavirus Disease-19 OR 2019 Novel Coronavirus Disease	
**Control**		
**Outcome**	Treatment OR Therapy changes OR Treatment Switching OR Therapeutics OR Therapy OR Consequencies OR Disease progression OR Disease Flare OR Symptom Flare Up OR Conservative Treatment OR Pharmaceutical Preparations OR Musculoskeletal Manipulations OR Drug Therapy OR antirheumatic agent OR disease modifying antirheumatic drug OR Route Of Administration OR Orthopedic Procedures OR Replacement Arthroplasty OR Replacement Hip OR Replacement Knee OR Rehabilitation	Therapy OR disease exacerbation OR Treatment Switching OR conservative treatment OR drug therapy OR OR antirheumatic agent OR disease modifying antirheumatic drug OR orthopedic surgery OR replacement arthroplasty
**Method**	observational studies, Randomised controled trials, Cohort-Studies	

<u>Applied Filters on Embase:</u>

- Date: 2019- 2021
- Languages: German and English
- Age: 19- 64 years, 65 years and older, 80 years and older

Search-terms on Pubmed and Cochrane:

	All fields	Mesh terms
**Patient**	Musculoskeletal Diseases OR Musculoskeletal Disorders OR Bone Diseases OR Fasziitis Plantaris OR Joint Diseases OR Arthritis OR Halux Rigidus OR Joint Deformities OR Synovitis OR Muscular Diseases OR Contracture OR Fibromyalgia OR Chronic Fatigue Syndrom OR Muscular Disorders OR Myofascial Pain Syndromes OR Myopathies OR Myositis OR Inclusion Body Myositis OR Polymyositis OR Dermatomyositis OR Polymyalgia Rheumatica OR Tendinopathy OR Rheumatoid Arthritis OR Crystal Arthropathies OR Gout OR Chondrocalcinosis OR Osteoarthritis OR Rheumatic Fever OR Sjogren's syndrome OR Adult-onset Still's Disease OR Felty Syndrom OR Vasculitis OR Aortitis OR Arteritis OR Giant Cell Arteritis OR Polyarteritis Nodosa OR Takayasu Arteritis OR Behcet Syndrom OR Purpura Schoenlein-Henoch OR Anti-Neutrophil Cytoplasmic Antibody-Associated Vasculitis OR Churg-Strauss Syndrom OR Granulomatosis With Polyangiitis OR Microscopic Polyangiitis OR Rheumatoid Vasculitis OR Thromboangiitis Obliterans OR Cutaneous Leukocytoclastic Vasculitis OR Spondylarthritis OR Psoriatic Arthritis OR Reactive Arthritis OR Ankylosing Spondylitis OR Inflammatory Bowel Disease-Associated Arthritis OR Infectious Arthritis OR Whipple Disease OR Hereditary Autoinflammatory Diseases OR Cryopyrin-Asssociated Periodic	Musculoskeletal Diseases OR Bone Diseases OR Fasziitis Plantaris OR Joint Diseases OR Arthritis OR Halux Rigidus OR Joint Deformities OR Synovitis OR Muscular Diseases OR Contracture OR Fibromyalgia OR Chronic Fatigue Syndrom OR Muscular Disorders OR Myofascial Pain Syndromes OR Myopathies OR Myositis OR (Myositis, Inclusion Body), Polymyositis OR Dermatomyositis OR Polymyalgia Rheumatica OR Tendinopathy OR (Arthritis, Rheumatoid) OR Crystal Arthropathies OR Gout OR Chondrocalcinosis OR Osteoarthritis OR Rheumatic Fever OR Sjogren's syndrome OR (Still's Disease, Adult-onset) OR Felty Syndrom OR Vasculitis OR Aortitis OR Arteritis OR Giant Cell Arteritis OR Polyarteritis Nodosa OR Takayasu Arteritis OR Behcet Syndrom OR (Purpura, Schoenlein-Henoch) OR Anti-Neutrophil Cytoplasmic Antibody-Associated Vasculitis OR Churg-Strauss Syndrom OR Granulomatosis With Polyangiitis OR Microscopic Polyangiitis OR Rheumatoid Vasculitis OR Thromboangiitis Obliterans OR (Vasculitis, Leukocytoclastic, Cutaneous) OR Spondylarthritis OR (Arhritis, Psoriatic) OR (Arthritis, Reactive) OR (Spondylitis, Ankylosing) OR (Arthritis, Infectious) OR Whipple Disease OR Hereditary Autoinflammatory Diseases OR Cryopyrin-Asssociated Periodic Syndrome OR Familial Mediterranean

	Syndrome OR Familial Mediterranean Fever OR systemic scleroderma OR diffuse scleroderma OR Sarcoidosis OR Antiphospholipid Syndrom OR Undifferentiated Connective Tissue Diseases OR Antisynthetase Syndrom OR Anti-Glomerular Basement Membrane Disease OR Systemic Lupus Erythematosus OR Osteoporosis OR Bone Density	Fever OR (Scleroderma, Systemic) OR (Scleroderma, Diffuse) OR (Scleroderma, Limited) OR SarcoidosisOR Antiphospholipid Syndrom OR Undifferentiated Connective Tissue Diseases OR Antisynthetase Syndrom OR Anti-Glomerular Basement Membrane Disease OR (Lupus Erythematosus, Systemic) OR osteoporosis OR bone density
**Intervention**	COVID-19 OR Severe Acute Respiratory Syndrome Coronavirus 2 OR SARS-CoV2 OR COVID-19 pandemic OR Novel coronavirus pneumonia OR SARS-CoV2-Infection OR COVID-19 Virus Disease OR COVID-19 Virus Infection OR Coronavirus Disease-19 OR 2019 Novel Coronavirus Disease	COVID-19, SARS-CoV-2
**Control**		
**Outcome**	Treatment OR Therapy changes OR Treatment Switching OR Therapeutics OR Therapy OR Consequencies OR Disease progression OR Disease Flare OR Symptom Flare Up OR Conservative Treatment OR Pharmaceutical Preparations OR Musculoskeletal Manipulations OR Drug Therapy OR Molecular Targeted Therapy OR Route Of Administration OR Orthopedic Procedures OR Replacement Arthroplasty OR Replacement Hip OR Replacement Knee OR Rehabilitation	Therapeutics, therapy, Treatment Outcome, Treatment Switching, Conservative Treatment, COVID-19 drug treatment, Supplementary Concept, Symptom Flare Up, Pharmaceutical Preparations, Musculoskeletal Manipulations, Drug Therapy, Molecular Targeted Therapy, Orthopedic Procedures, Arthroplasty, Replacement, (Arthroplasty, Replacement, Hip), (Arthroplasty, Replacement, Knee), Rehabilitation
**Method**	observational studies, Randomised controled trials, Cohort-Studies	

Applied filters on Cochrane:

- "Last two years"

Applied filters on Pubmed

- Languages: German and English
- Population: Humans
- Time: 12/2019 – 10/2021
- Age: 19+
